# Effect of Hydrogen Peroxide Bleaching on Color Stability and Microhardness of Alkasite Restorative Materials: An In Vitro Study

**DOI:** 10.7759/cureus.91016

**Published:** 2025-08-26

**Authors:** Souad A Alfouzan, Rahaf A Alolayan, Asma Munir Khan

**Affiliations:** 1 Restorative Dentistry, The One Clinic, Buraidah, SAU; 2 Pediatric Dentistry, Dr. Tooth Clinic, Unaizah, SAU; 3 Operative Dentistry, Qassim University, Buraidah, SAU

**Keywords:** alkasite, bleaching agents, cention n, color stability, hydrogen peroxide, microhardness

## Abstract

Introduction: High-concentration hydrogen peroxide (HP) bleaching agents are widely used in dental practice but may compromise the optical and mechanical properties of restorative materials. This in vitro study evaluated the impact of 40% HP bleaching on the color stability and microhardness of alkasite restorative material under different curing modes.

Methods: Sixty discs of alkasite restoration (Cention N) were prepared and divided into two groups: chemical-cured (n=30) and dual light-cured (n=30). Color coordinates (L*, a*, b*) were measured using spectrophotometry, and microhardness was assessed using Vickers testing before and after three consecutive 20-minute bleaching sessions with 40% HP. Data were analyzed using paired t-tests and one-way analysis of variance (ANOVA) with post-hoc Tukey tests (P < 0.05).

Results: Bleaching significantly altered alkasite color with an overall change of ΔE = 1.77 ± 1.45 (P < 0.001), exceeding the clinically acceptable threshold of ΔE = 1.0. Chemical-cured specimens showed significantly greater color change (ΔE = 2.22 ± 1.81) than light-cured specimens (ΔE = 1.31 ± 0.74, P = 0.015). Microhardness decreased significantly in both groups after bleaching (P < 0.001), with chemical-cured specimens exhibiting greater reduction.

Conclusions: High-concentration HP bleaching significantly compromises both color stability and microhardness of alkasite restorations, with chemical-cured specimens being more susceptible to these changes than light-cured specimens. These findings suggest that high-concentration bleaching may adversely affect alkasite restoration longevity and aesthetic outcomes.

## Introduction

The use of peroxide bleaching agents has significantly increased in recent years as a means to improve the aesthetic appearance of natural dentition. These agents, typically composed of hydrogen peroxide (HP) or carbamide peroxide (CP), work by producing free radicals that break down pigmented molecules, resulting in a whitening effect [1,2]. However, exposure to bleaching agents can also affect restorative materials, potentially altering their optical, physical, and chemical properties [3,4].

Cention N is an alkasite-based, tooth-colored restorative material that has been introduced as a fluoride-releasing material with both self-cure and optional light-cure properties [5]. This bioactive material combines the benefits of glass ionomer cements and composite resins, offering fluoride release, alkaline pH buffering, and acceptable mechanical properties [6]. The dual-cure mechanism of Cention N allows flexibility in clinical applications, particularly in situations where light access is limited [7].

Prior research indicates that bleaching agents can cause oxidation of resin matrices, resulting in color alterations and potential deterioration of mechanical properties [8,9]. Studies suggest that the effects of bleaching may vary based on the material composition and the specific protocols employed [10-12]. The extent of polymerization achieved via various curing methods may influence the material's susceptibility to bleaching-induced alterations. Light-cured materials generally achieve greater degrees of conversion than chemically cured materials, potentially leading to different responses to oxidative challenges from HP [13-15].

Recent investigations have indicated that HP bleaching may decrease the flexural strength of Cention N regardless of polymerization mode [16,17], and high-concentration HP significantly reduces microhardness in composite materials [18-20]. Furthermore, studies have demonstrated that bleaching agents can affect the surface morphology and chemical composition of various restorative materials [21,22].

Limited data is available regarding the impact of bleaching agents on the color stability and microhardness of Cention N, particularly comparing different curing modes. This study aimed to evaluate the effect of 40% HP bleaching on the color stability and microhardness of Cention N restorative material under different curing modes.

## Materials and methods

Study design

This experimental in vitro study was designed as a controlled laboratory investigation to assess the effects of HP bleaching on alkasite restorative material properties under standardized conditions. The study protocol was approved by the Ethics Committee of the College of Dentistry, Qassim University (EA/6069/2021).

Specimen preparation

Sixty disk-shaped specimens (10 mm diameter × 2 mm thickness) of Cention N (Ivoclar Vivadent, Schaan, Liechtenstein) were prepared using a custom-made stainless steel mold. The specimens were divided into two groups based on curing methodology. The first group consisted of chemical-cured specimens where the material was mixed according to the manufacturer's instructions and allowed to cure chemically without light activation for five minutes at room temperature. The second group comprised dual light-cured specimens where the material was mixed according to the manufacturer's instructions and immediately light-cured using an LED light-curing unit (Elipar DeepCure-S, 3M ESPE, St. Paul, MN, USA) at 400 mW/cm^2^ intensity, which was verified with a calibrated radiometer (Cure Rite, DENTSPLY Caulk, Milford, DE, USA). The light-cured specimens received 40 seconds of exposure through a transparent matrix, followed by an additional 20 seconds after removal from the mold to ensure complete polymerization of all surfaces.

All specimens were finished with 600-grit silicon carbide paper using a MetaServ 250 Grinder-Polisher with Vector Power Head (Buehler, USA) under water cooling to achieve a uniform surface texture and remove any excess material. Following preparation, all specimens were stored in distilled water at 37°C for 24 hours to allow complete polymerization and eliminate uncured monomers. Subsequently, specimens were stored in light-proof containers at room temperature until testing commenced.

Color measurement

Color measurements were recorded using a calibrated spectrophotometer (Color-Eye 7000A, GretagMacbeth, New Windsor, NY, USA) under standardized lighting conditions using D65 illuminant with a 10° standard observer. The instrument was calibrated using standard white and black tiles before each measurement session to ensure accuracy. The Commission Internationale de l’Éclairage (CIE) Lab* color system was employed, measuring L* (lightness, 0-100), a* (red-green axis, -60 to +60), and b* (yellow-blue axis, -60 to +60) coordinates.

To minimize measurement errors, three measurements were taken at different locations on each specimen surface and subsequently averaged. The same operator performed all measurements to ensure consistency throughout the study. Color difference (ΔE) was calculated using the CIE 1976 formula: $\Delta E = \sqrt{ (\Delta L^*)^2 + (\Delta a^*)^2 + (\Delta b^*)^2 }$ where Δ represents the difference between baseline and post-bleaching values.

Bleaching procedure

The bleaching protocol was standardized and performed under controlled conditions using 40% HP gel (Opalescence Boost, Ultradent Products Inc., South Jordan, UT, USA) following the manufacturer's instructions. Three consecutive bleaching sessions were performed with a standardized protocol where bleaching gel was applied uniformly to the specimen surface using a disposable applicator tip in a 1.0 mm thick layer, ensuring complete surface coverage. Each specimen was bleached for 20 minutes per session, after which specimens were thoroughly rinsed with distilled water for 30 seconds and gently dried with oil-free compressed air. Fresh bleaching gel was applied for subsequent sessions following the same methodology. After the final session, specimens were stored in fresh distilled water at 37°C for 24 hours before post-bleaching measurements were obtained.

Microhardness testing

Microhardness was assessed using the Vickers hardness test with a NOVA 130 microhardness tester (Innovatest Europe BV, Maastricht, Netherlands) equipped with a diamond pyramid indenter. Testing parameters were standardized with a 200 g load (1.96 N) applied for a 15-second dwell time. This load was selected based on preliminary testing to ensure adequate indentation size without causing specimen damage.

Five indentations were made on each specimen surface following a predetermined pattern while maintaining a minimum distance of 100 μm between indentations to avoid interference effects. Measurements were performed before and after the complete bleaching protocol under 400× magnification by the same trained operator using the integrated measurement software. The Vickers hardness number (VHN) was automatically calculated by the software based on diagonal measurements of the indentations.

Statistical analysis

Statistical analysis was performed using IBM SPSS Statistics for Windows, Version 24 (Released 2017; IBM Corp., Armonk, New York, United States). The normality of data distribution was assessed using the Shapiro-Wilk test, and descriptive statistics included means, standard deviations, and 95% confidence intervals. For color parameters and microhardness values, comparisons were made between pre- and post-bleaching measurements within each group using paired t-tests, between curing modes using independent t-tests, and multiple group comparisons using one-way analysis of variance (ANOVA) followed by post-hoc Tukey honestly significant difference (HSD) tests. Statistical significance was set at P < 0.05.

## Results

Color stability analysis

Bleaching treatment caused statistically significant color changes in Cention N specimens. The overall color change analysis revealed a significant alteration (ΔE = 1.77 ± 1.45, P < 0.001) following the complete bleaching protocol.

Comparison of curing modes on color change

Chemical-cured specimens exhibited significantly greater color change (ΔE = 2.22 ± 1.81) compared to light-cured specimens (ΔE = 1.31 ± 0.74), with statistical significance (P = 0.015) (Table 1).

**Table 1:** Comparison of curing modes on color change

Group	ΔE (Mean ± SD)	95% CI	P-value
Light-cured	1.31 ± 0.74	1.03-1.59	0.02
Chemical-cured	2.22 ± 1.81	1.54-2.90	0.02

Color coordinate analysis

Individual color coordinate analysis showed distinct patterns for each curing mode. Chemical-cured specimens demonstrated L* increase from 59.40 ± 2.77 to 60.07 ± 1.63 (P = 0.067), a* change from -1.20 ± 0.09 to -1.04 ± 0.22 (P < 0.001), and b* change from 4.43 ± 0.67 to 4.46 ± 0.24 (P = 0.803).

Light-cured specimens showed L* increase from 59.84 ± 0.87 to 60.64 ± 0.63 (P < 0.001), a* change from -1.10 ± 0.17 to -0.97 ± 0.03 (P < 0.001), and b* decrease from 4.37 ± 0.73 to 4.10 ± 0.53 (P = 0.043) (Table 2).

**Table 2:** Color coordinates before and after bleaching

Group	L* Before	L* After	a* Before	a* After	b* Before	b* After
Chemical-cured	59.40 ± 2.77	60.07 ± 1.63	-1.20 ± 0.09	-1.04 ± 0.22	4.43 ± 0.67	4.46 ± 0.24
Light-cured	59.84 ± 0.87	60.64 ± 0.63	-1.10 ± 0.17	-0.97 ± 0.03	4.37 ± 0.73	4.10 ± 0.53

Microhardness analysis

Microhardness measurements revealed a significant reduction following bleaching treatment. The overall microhardness decreased significantly from 41.13 ± 1.99 to 39.88 ± 2.18 (P < 0.001).

One-way ANOVA revealed significant differences between all groups (P < 0.001). Light-cured specimens maintained higher microhardness values both before (42.87 ± 0.74 VHN) and after (41.88 ± 0.75 VHN) bleaching compared to chemical-cured specimens (39.38 ± 1.12 VHN before and 37.88 ± 0.93 VHN after bleaching). Post-hoc Tukey tests confirmed significant differences between all tested subgroups (P < 0.001 for all comparisons) (Table 3).

**Table 3:** Effect of curing mode on microhardness VHN: Vickers hardness number

Group	Condition	Microhardness (VHN)	95% CI	P-value
Light-cured	Before	42.87 ± 0.74	42.59-43.15	<0.05
Light-cured	After	41.88 ± 0.75	41.60-42.16	<0.05
Chemical-cured	Before	39.38 ± 1.12	38.96-39.80	<0.05
Chemical-cured	After	37.88 ± 0.93	37.53-38.23	<0.05

## Discussion

The present study demonstrated that 40% HP bleaching significantly affected both the color stability and microhardness of Cention N restorative material. The overall color change (ΔE = 1.77) exceeded the clinically acceptable threshold of ΔE = 1.0, suggesting that the color alteration might be perceptible to the human eye and potentially clinically relevant under the tested conditions [23]. This finding has important implications for clinical practice, as it indicates that high-concentration bleaching treatments may compromise the aesthetic properties of Cention N restorations.

The current findings align well with existing research on bleaching effects on restorative materials. Sajini (2025) reported noticeable color change in Cention N after bleaching, though less pronounced than in other materials [16]. Similarly, Ahmed et al. (2025) demonstrated that bleaching agents can affect the degradation resistance of next-generation dental composites [17]. These results are consistent with previous investigations by Fernandes et al. (2020) and Yu et al. (2018), who reported significant effects of high-concentration HP on composite microhardness and physical properties [18,10]. Earlier work by Turker and Biskin (2003) and Bailey and Swift (1992) similarly reported varying responses of restorative materials to bleaching agents, with polymer structure and curing method being important factors [9,24].

An important finding of this study was the differential response between chemical-cured and light-cured specimens. Chemical-cured specimens appeared more susceptible to color changes compared to light-cured specimens, which might be attributed to differences in polymerization degree and residual monomer content. Light-cured specimens likely achieved a higher degree of conversion, potentially resulting in a more cross-linked polymer network that could offer greater resistance to oxidative degradation caused by HP [25].

The potentially lower degree of conversion in chemical-cured specimens may leave more unreacted monomers and create a less dense polymer structure, possibly making the material more permeable to HP penetration [26]. This finding suggests that the curing method significantly influences the material's resistance to bleaching-induced changes, which has practical implications for restoration placement and patient care.

The observed microhardness reduction in both curing modes suggests that HP may cause polymer chain scission and plasticization effects, potentially leading to reduced cross-link density [26,27]. This degradation mechanism is consistent with the known oxidative effects of HP on polymer-based dental materials and explains why both aesthetic and mechanical properties were compromised in the present study.

These findings suggest that clinicians should exercise caution when performing high-concentration HP bleaching in patients with Cention N restorations, particularly those that are chemically cured.

The findings of this in vitro study should be interpreted with caution, as laboratory conditions may not fully replicate the complex oral environment, including saliva buffering, temperature fluctuations, and concurrent chemical exposures that could influence material behavior.

Future research should explore different bleaching protocols, investigate recovery mechanisms, and assess the clinical performance of bleached Cention N restorations over extended periods. Long-term studies evaluating material behavior under physiological conditions would provide valuable insights into the clinical relevance of these findings.

## Conclusions

Under the conditions of this in vitro study, 40% HP bleaching significantly altered the color stability and reduced the microhardness of Cention N restorative material. Chemical-cured specimens demonstrated greater susceptibility to both color change and microhardness reduction compared to light-cured specimens, suggesting that light curing may provide enhanced resistance to bleaching-induced degradation. These findings have important clinical implications for treatment planning and patient care when bleaching procedures are considered in the presence of Cention N restorations.
